# What to Know About Falls in Older Adults? Risk Factors, Predictors, and Therapeutic Interventions

**DOI:** 10.3390/ijerph22121863

**Published:** 2025-12-14

**Authors:** Fernanda Bueno Pilastri, Julia Fantim Lopez, Eric Nkansah Boateng, Nise Ribeiro Marques

**Affiliations:** 1 Faculty of Philosophy and Science, São Paulo State University, São Paulo 17525-900, Brazil; 2 School of Enginnering, Lovely Professional University, Jalandhar 144411, India

**Keywords:** gerontology, physical therapy, prevention

## Abstract

Background: Approximately one quarter of community-dwelling older adults experience at least one fall each year. Falls can result in soft tissue injuries, fractures, or even death. Given this high prevalence, it is essential to identify fall-related risk factors, develop predictive models, and prescribe effective exercise-based interventions to prevent falls. Objective: To analyze risk factors, predictors, and therapeutic interventions for falls in older adults. Methods: A literature search was conducted in SCIELO, PUBMED, and PEDro databases between 15–20 October 2025. Inclusion criteria comprised peer-reviewed, open-access studies in English published from 2020 onward. Findings were categorized into three domains: (1) fall risk factors, (2) predictive models, and (3) exercise-based interventions. Twenty studies met the inclusion criteria. Results: Falls among older adults arise from multifactorial interactions involving physical, clinical, cognitive, and social factors such as impaired mobility, comorbidities, polypharmacy, and cognitive decline. Lower-limb strength and functional performance are key determinants of fall risk. Current predictive models show limited accuracy, with fall history as the strongest predictor. Exercise-based interventions, particularly multicomponent and home-based programs, improve balance, strength, and mobility but show variable effects on fall rates. The absence of standardized parameters for exercise prescription limits the development of evidence-based guidelines. Conclusions: Falls in older adults are multifactorial events influenced by physical and cognitive decline. Predictive models remain imprecise, and although exercise interventions improve functional outcomes, their impact on reducing falls is inconsistent. Standardized exercise protocols are needed to optimize fall prevention strategies.

## 1. Introduction

Falls among older adults represent significant adverse events that can result in musculoskeletal injuries, hospitalization, psychological consequences such as fear of falling, and an increased risk of mortality [1]. It is estimated that every year, more than one-quarter of older adults experience at least one fall. Although most falls are non-lethal, it is common for older adults to require some form of healthcare assistance following a fall episode. Falls are considered a major public health concern worldwide, as they are among the leading causes of injury-related morbidity and mortality in the older adult population [2]. Furthermore, the consequences of falls extend beyond physical harm, contributing to loss of independence, reduced quality of life, and increased healthcare costs [3].

Age-related physical impairments, such as loss of strength, balance dysfunction, decline in muscle power, and visual and cognitive deficits, have been identified as factors that increase the risk of falls [4]. In addition, psychological and sociodemographic factors, comorbidities, and medication use have also been consistently reported in the literature as contributing risk factors for falls among older adults. Thus, it is suggested that fall risk results from a complex interplay of multiple determinants, and the predictive models capable of accurately identifying and preventing falls in this population remain insufficiently established [1].

Consistent with the current limitations observed in fall-prediction methodologies, there remains no clear consensus in the scientific literature regarding the most effective exercise-based therapeutic strategies for falls prevention in older adults [3]. Although multimodal exercise interventions incorporating aerobic and resistance training, as well as unimodal programs focused on isolated modalities such as resistance, aerobic, or balance training, gait training, Pilates, and Tai Chi, have been widely investigated, their effects on modifiable falls-risk determinants have generally ranged from minimal to moderate. Importantly, a persistent methodological gap in literature concerns the absence of standardized parameters for exercise prescription, particularly with respect to training volume, intensity, and progression [5]. This lack of uniformity may partly explain the variability in outcomes and hinders the establishment of evidence-based exercise guidelines for fall prevention in the older adult population.

Falls represent a critical threat to healthy ageing, contributing to injury, functional decline, and loss of independence in older adults [6]. Although numerous biological, psychological, and environmental factors associated with fall risk have been described, predictive accuracy remains limited and clinically actionable models are still lacking. Exercise-based interventions are widely advocated as a cornerstone of fall prevention; however, heterogeneity in program design and insufficient standardization of key training parameters have resulted in variable outcomes and uncertainty regarding best-practice recommendations [3]. Therefore, synthesizing current evidence on fall-related risk factors, predictive markers, and therapeutic strategies is essential to inform clinical practice and advance precision approaches to fall prevention in older adults. Thus, this scoping review aimed to map and analyze the evidence on risk factors, predictors, and therapeutic interventions for falls in older adults, identifying key concepts, evidence gaps, and the characteristics of available studies. To guide the review process and enhance analytical coherence, the following research questions were established: (1) What risk factors for falls have been reported in older adults? (2) What predictors or assessment tools are used to estimate fall risk? (3) What exercise therapeutic interventions and related effects are described for fall prevention?

## 2. Methods

A multidisciplinary research team with expertise in gerontology, rehabilitation sciences, and quantitative methods was formed to conduct this review. The review followed the PRISMA-ScR guidelines to map the scope and nature of the existing evidence on factors associated with falls in older adults (Table 1). A scoping review approach was chosen to capture the breadth of research addressing risk factors, fall prediction models, and exercise-based strategies for fall prevention rather than to evaluate intervention effectiveness or conduct meta-analysis. This scoping review was registered on Open Science Framework (Registration DOI: 10.17605/OSF.IO/DM2RV), as it was designed to provide an exploratory mapping of recent evidence rather than a hypothesis-driven or quantitative synthesis. All methods were defined a priori to ensure transparency and reproducibility.

Eligibility criteria, search procedures, study selection, and data charting were defined a priori. Studies were screened in two stages: title/abstract and full text by independent reviewers, with disagreements resolved through consensus or third-reviewer adjudication. To reflect the multidimensional nature of falls, findings were organized into three thematic domains: (1) risk factors for falls; (2) models used to predict fall risk; and (3) exercise and rehabilitation programs designed to prevent falls in older adults. Data from each included study were extracted using a standardized charting form developed by the research team. The form captured key variables such as author, year of publication, country, study design, population characteristics, sample size, objectives, main outcomes, and principal findings relevant to fall risk factors, prediction models, or exercise interventions.

Eligible studies included randomized controlled trials, cohort studies, systematic reviews, and meta-analyses that investigated physical exercise or physical therapy interventions, as well as predictors related to functional capacity or physical performance associated with fall risk. Studies were required to involve community-dwelling older adults, including those with conditions such as Parkinson’s disease, stroke, or dementia, provided participants were not institutionalized.

Studies were excluded if they examined exclusively pharmacological, nutritional, or psychological interventions, or if they recruited institutionalized or hospitalized older adults. Research focused primarily on populations with frailty syndromes, cancer, diabetes, or orthopedic and musculoskeletal conditions such as hip fracture, osteoarthritis, or osteoporosis was also excluded, as these conditions alter fall risk profiles in ways that are not representative of community-dwelling aging. Additional reasons for exclusion included the absence of peer review, the inclusion of participants younger than 60 years, or the lack of outcomes related to falls or fall risk.

The literature search was conducted across SCIELO, PUBMED, and PEDro (Physiotherapy Evidence Database) databases. PubMed and PEDro were selected for their strong coverage of biomedical and rehabilitation research, while SciELO was included to capture studies from Latin America and the Global South, which are underrepresented in major international databases. This targeted approach is consistent with methodological guidance for scoping reviews [7,8]. The search was conducted between 15 and 20 October 2025. Inclusion criteria were restricted to peer-reviewed, open-access manuscripts published in English from 2020 onward, a timeframe selected to capture contemporary evidence that reflects current exercise prescription standards, recent advances in fall-risk predictive modeling, and post-pandemic shifts in fall-prevention practices among community-dwelling older adults.

The search strategy combined controlled vocabulary (e.g., MeSH terms) and free-text keywords related to falls, fall prediction, risk factors, fall prevention, physical exercise, and physical therapy. Boolean operators and truncation were used to refine and broaden the search (e.g., “falls” OR “fall risk” OR “fall prevention” AND “older adults” OR “elderly” AND “physical exercise” OR “physical therapy” OR “rehabilitation”; Table 2 shows SCIELO, PUBMED, and PEDro search stings).

After removing duplicates, titles and abstracts were screened independently by two reviewers to determine eligibility. Full-text screening was then performed for studies that met the initial criteria or lacked sufficient information at the abstract stage. Studies were excluded if they did not focus on older adults, did not address risk factors, predictors, or exercise-based interventions, or were not empirical research articles.

**Figure 1:**
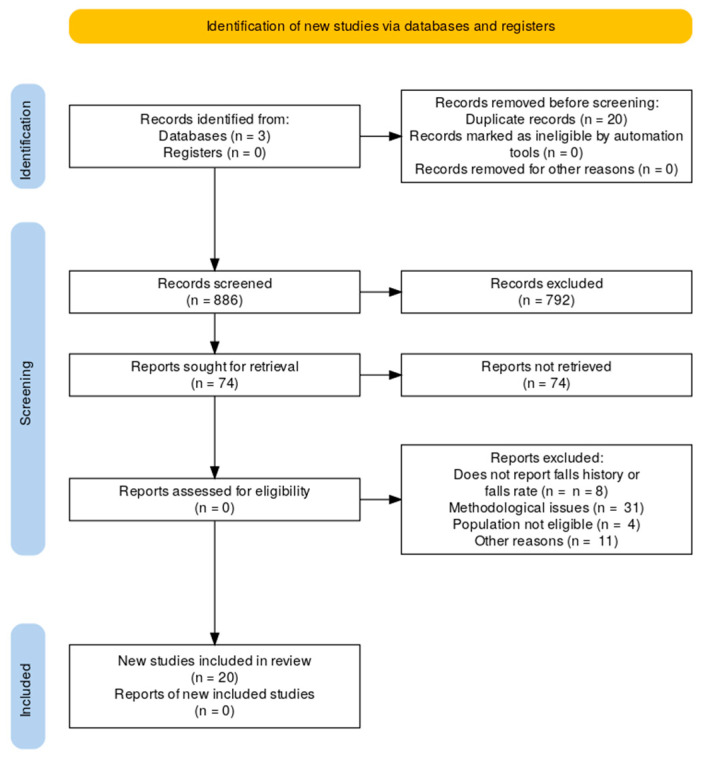
PRISMA flow chart showing the study selection process, including database search results, screening, eligibility assessment, and the final number of studies included in the review.

All screening steps, along with the number of records included and excluded at each stage and the reasons for exclusion, are presented in the PRISMA flow diagram (Figure 1). No formal quality assessment or risk of bias evaluation was conducted, as this scoping review aimed to provide an overview and mapping of the existing evidence rather than to appraise the methodological quality of individual studies.

## 3. Results

In total, as shown in Figure 1, 886 papers were retrieved from the three electronic databases searched. After removing 20 duplicates, 866 records were screened. Of these, 792 were excluded based on title, abstract, and keywords. The remaining 74 papers were assessed in full text. A total of 54 studies were excluded due to methodological issues (*n* = 31), population not eligible (*n* = 4), not reporting falls history (*n* = 8), or other reasons (*n* = 11). Finally, 20 studies were included in the review.

### 3.1. Synthesis of Results

The findings from the included studies were systematically collated and organized according to thematic domains that emerged from the literature. This approach enabled the integration of heterogeneous study designs and analytical frameworks while preserving the complexity inherent to falls research in older adults. Themes were identified inductively and structured to reflect the multifactorial determinants of fall risk (*n* = 6), the methodological approaches employed to predict falls (*n* = 2), and the exercise-based strategies implemented for fall prevention (*n* = 12). This thematic synthesis provides a comprehensive and conceptually coherent representation of the current evidence base, allowing for a multidimensional interpretation of factors associated with falls and the interventions designed to mitigate them among older adults.

### 3.2. Fall Risk Factors

All studies included in this scoping review defined a fall as an unexpected event in which an individual comes to rest on the ground, floor, or a lower level, not resulting from a major intrinsic event (such as stroke or syncope) or from an overwhelming external force. Among the six studies that examined risk factors for falls, only two stratified participants according to fall-related outcomes and the number of falls within a specified time frame, being the most commonly the year preceding the study. Both studies analyzed fall risk factors across different subgroups, including any fall, recurrent falls, injurious falls, and falls occurring indoors or outdoo Saunders and Xu [9,10] identified multiple interrelated domains of risk factors for falls among community-dwelling older adults. Physical factors included impaired mobility, balance impairment, poor gait performance (e.g., slow gait speed), low physical activity and functional capacity, reduced range of motion, malnutrition, and frailty. Clinical and behavioral factors encompassed chronic comorbidities such as cardiovascular disease, hypertension, and Parkinson’s disease, as well as polypharmacy, smoking, alcohol consumption, and fear of falling. Social factors, including single marital status and rural residence, were also associated with an increased likelihood of falls.

Complementing these findings, Zhu [5] conducted a systematic review and meta-analysis examining the association between lower limb strength parameters and falls in community-dwelling older adults. The main strength-related parameters investigated as fall risk factors were muscle power, rate of force development, rate of torque development, and peak joint torque. The joint torques most frequently assessed in previous studies included those of the hip and knee flexors and extensors, as well as ankle dorsiflexors and plantar flexors. Among these, knee extensor torque showed the strongest association with age-related strength loss and mobility decline. However, there is still no consensus regarding the extent to which reductions in joint torque, power, or rate of torque development contribute to increased fall risk in older adults.

Other studies have identified additional risk factors associated with sensory, cognitive, and psychological decline. Advanced age (over 80 years), reduced or uncorrected vision, depression, and cognitive decline were reported as significant contributors to fall risk [11,12]. In particular, impairments in executive function appear to play a major role in fall susceptibility, and the Trail Making Test has been suggested as a useful tool to assess executive performance related to falls in older adults [12].

Building on this evidence, Hsieh and Jiang [13] highlighted the role of additional physical and behavioral factors, including sedentary lifestyle, sarcopenia, bone mass loss, and sensory impairments, as significant contributors to fall risk. These conditions exacerbate functional decline by reducing muscle strength, balance control, and postural stability, thereby increasing vulnerability to falls and complementing the evidence linking cognitive, behavioral, and physical performance to fall risk.

Finally, regarding functional capacity assessments, previous studies have consistently shown that poor performance on standardized tests is associated with an elevated risk of falls in older adults. Specifically, a Short Physical Performance Battery (SPPB) score below 10 points, a gait speed of less than 0.8 m·s^−1^, and a Timed Up and Go completion time greater than 12 s have been identified as strong predictors of fall risk [11,14]. Table 3 synthesizes these findings.

### 3.3. Prediction Models of Falls in Older Adults

Fall prevention interventions are most effective when directed toward older adults at high risk of falls. However, given the multifactorial nature of falls, accurately identifying those at greatest risk remains challenging. Prognostic models aim to integrate multiple risk factors to estimate an individual’s risk of future falls [15]. The systematic review by [15] was the first to comprehensively summarize falls prediction models for community-dwelling older adults, identifying 72 models in total. Most of these models exhibited a high risk of bias, primarily due to methodological limitations in statistical analyses, outcome assessments, and restrictive eligibility criteria. Consequently, the authors concluded that the clinical applicability of these models remains limited, as their predictive performance is largely unreliable.

Wapp [16] proposed a validation of a fall rate prediction model for community-dwelling older adults combining three cohort studies and considering data of 1850 older people. All three predictive models identified the number of prior falls in the past 12 months as the main predictor of future falls. According to this, Wapp [16] suggested that the number of prior falls is a robust predictor for future falls in community-dwelling older adults. The authors also identified that variables such as physical performance tests, age, sex, comorbidities, medication, or quality of life were not improving the predictive accuracy of the models when it is combined with the history of falls.

Gade and Wapp [15,16] suggested that the main challenge in developing and validating fall prediction models for older adults lies in the complex and multifactorial nature of falls, which result from diverse combinations of individual risk factors. Because these interactions are difficult to capture statistically, a history of prior falls may serve as the most comprehensive indicator of overall fall risk [16]. However, relying solely on fall history limits a model’s ability to identify first-time fallers, as individuals without previous falls receive identical predicted risks. Therefore, future models should incorporate additional sensitive predictors to better detect those at risk of experiencing their first fall. In this regard, the most frequently reported risk factors in the literature include fall history, age, gait parameters, comorbidities (e.g., cardiovascular disease, hypertension, Parkinson’s disease, and frailty), functional impairments, polypharmacy, cognitive status (including depression and fear of falling), and lifestyle-related factors such as sedentary behavior, alcohol consumption, and smoking [9,10,11,12,13].

### 3.4. Exercise Interventions to Prevent Falls

Exercise intervention is considered one of the most effective approaches to prevent falls in the older population [3]. The benefits of exercise programs are primarily associated with improvements in physical performance, muscle strength, balance, and aerobic capacity, which collectively help reduce early fatigue and fall risk in older adults [17]. Moreover, regular physical exercise acts as a protective factor against cardiovascular comorbidities, frailty, and cognitive decline [3]. However, despite the well-established benefits of exercise, current guideline protocols lack clear standardization for exercise prescription, particularly regarding training volume, intensity, and progression [5]. Consequently, in recent years, several exercise protocols have been proposed to enhance fall prevention strategies among older adults.

Kasicki [18] systematic review assessed whether multicomponent training programs combining strength, balance, and aerobic exercises are more effective at preventing falls in older adults than single-component exercise programs. This study analyzed data of functional outcomes, physiological measures and falls history of six randomized controlled trials (RCT). According to the findings of this study, multicomponent training and single component training had similar effects in physical function and physiological parameters associated with falls risk. Also, the results about falls prevalence are inconclusive.

After the COVID-19 pandemic, unsupervised home-based exercise programs became a common approach to maintaining an active lifestyle and promoting healthy aging. Accordingly, Zhou [14] investigated the effectiveness, safety, and adherence of home-based strength and balance exercises for fall prevention among community-dwelling older adults. The study found that after a 12-month intervention, the risk of new falls decreased significantly by 25.3% in the group that performed the home-based exercise program. However, no statistically significant difference was observed in the overall rate of new falls between the home-based exercise group and the control group, which only received guidance and continued with their usual daily activities.

The meta-analysis by [19] examined the effectiveness of home-based exercise programs using electronic devices on fall-related outcomes among community-dwelling older adults. Twelve randomized controlled trials (RCTs) were included, assessing outcomes such as balance, lower-limb strength, fall risk, and fall efficacy. The analysis demonstrated that home-based exercise programs incorporating e-devices significantly reduced fall risk by 18% compared with controls. Moreover, the rate of falls decreased by 24–34%, depending on the specific type of exercise training protocol implemented.

Chen [20] conducted a systematic review and meta-analysis to quantitatively assess and compare the effects of traditional physical training, Tai Chi, multicomponent exercise programs, and exergame interventions on balance control and fall prevention in healthy older adults. Twenty studies with a total of 845 participants were included. Compared with the control groups, exergame interventions produced a greater reduction in fall rates.

Sievänen and Raikkonen [21,22] investigated the effects of whole-body vibration (WBV) training programs on fall reduction among community-dwelling older adults. Both studies implemented interventions consisting of at least 20 WBV training sessions. Despite differences in training protocols, both studies demonstrated improvements in physical function parameters; however, WBV training showed no significant effect in preventing falls among community-dwelling older adults.

Nørgaard [23] investigated the effectiveness of exercise on fall prevention in community-dwelling older adults. The exercise protocol consisted of six months of supervised weekly gym and Tai Chi sessions. A 14.3% fall rate reduction was detected in the exercise group compared with the control group. Also, the findings of this study demonstrated that there was reduction of 41% in falls with severe injury and pain in the exercise group.

Perturbation-based balance training (PBT) involves exposing individuals to repeated external disturbances in a controlled and safe environment to enhance their reactive balance responses. To investigate the effects of PBT on daily-life fall rates among community-dwelling older adults, Fernández-Rodríguez [24] conducted a study comparing a four-session treadmill-based PBT intervention with regular treadmill walking. The results of this randomized controlled trial, which included 140 high-functioning community-dwelling older adults, showed that PBT had no significant effect on reducing fall rates.

Długosz-Boś and Li [24,25] evaluated the effects of Pilates exercise on fall risk in older adults. The systematic review by [23,24] both suggested that Pilates training significantly improves fall-related outcomes. However, in both studies, fall risk was assessed using functional performance tests rather than actual fall incidence, which limits the generalizability of their findings regarding the effectiveness of Pilates in reducing fall risk among older adults.

**Table 4:** Summary of studies evaluating the effects of different exercise interventions on fall prevention among older adults [16,18,19,20,21,24,26,27,28].

Author(s) and Year	Study Type	Intervention	Duration	Main Findings	Conclusion on Fall Prevention
[18]	Systematic review	Multicomponent training vs. single-component training	Variable	Similar effects on physical function and physiological parameters; inconclusive results on fall prevalence	No significant difference between training types.
[14]	RCT	Home-based strength and balance exercise	12 months	Reduction in new fall risk; no significant difference in total fall rate vs. control	Partial efficacy
[19]	Meta-analysis	Home-based exercise using electronic devices	Variable	Reduction in risk and falls rate	Effective
[20]	Systematic review and meta-analysis	Traditional physical exercise training vs. exergame intervention	Variable	Exergames produced the greatest reduction in fall rates compared with traditional exercise training	Exergames most effective for fall prevention
[21]	RCTs	Whole-body vibration (WBV) training	≥20 sessions	No significant effect on falls rate	Ineffective
[16]	RCT	Supervised gym and Tai Chi sessions	6 months	Reduction in total falls and in severe injury-related falls	Effective
[24]	RCT	Perturbation-based balance training (PBT) vs. treadmill walking	4 sessions	No significant effect on falls rates	Ineffective
[26]	Systematic review and RCT	Pilates	Variable	Improvements in functional tests related to fall risk, but no direct falls incidence data	Limited evidence
[27]	Systematic review and meta-analysis	Older adults with and without cognitive impairment; various exercise types	Variable	Significant reduction in fall rates favoring exercise interventions	Effective
[28]	RCT	Aerobic-resistance and cognitive training in mild cognitive impairment	20 weeks	Reduced fall risk	Effective

**Figure 2:**
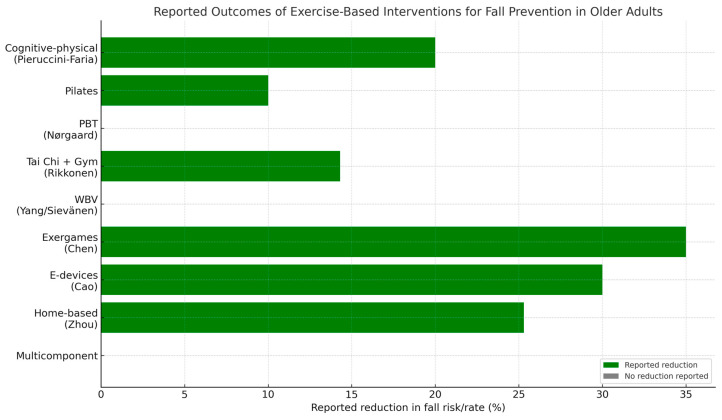
Reported outcomes of exercise-based interventions for fall prevention in older adults.

Pieruccini-Faria and Diamond [27,28] investigated the effects of exercise interventions on fall risk among older adults with cognitive impairments. The randomized controlled trial conducted by [25] demonstrated that 20 weeks of combined aerobic–resistance exercise and cognitive training significantly reduced fall risk in older adults with mild cognitive impairment (MCI). Vitamin D supplementation did not provide any additional benefits for fall prevention in this sample. The systematic review and meta-analysis by [26] found that exercise interventions incorporating various types of training protocols (e.g., resistance, aerobic, and balance exercises) resulted in a significant reduction in fall rates compared with control groups. Table 4 summarizes the main findings of these studies, and Figure 2 illustrates the efficacy of exercise-based interventions for fall prevention.

## 4. Discussion

The present review aimed to examine risk factors, predictors, and therapeutic interventions related to falls among community-dwelling older adults. This scoping review demonstrates that despite substantial research on falls in older adults, the field remains theoretically fragmented and methodologically inconsistent. Although numerous studies document associations between physical, cognitive, behavioral, and environmental risk factors, few integrate these determinants within coherent conceptual models capable of predicting falls, particularly for the first fall. The main challenges remain centered on accurately identifying the multifactorial determinants of falls and understanding how these interrelated factors can be systematically integrated into robust predictive models capable of effectively screening older adults at increased risk of falling. Despite growing scientific attention to this topic, there is still a lack of consensus regarding standardized methodologies for exercise prescription aimed at falls prevention, underscoring the need for clearer guidelines and harmonized protocols to optimize intervention efficacy and comparability across studies.

### 4.1. Risk Factors for Falls: A Multifactorial Phenomenon Requiring Integrated Models

The findings of the present scoping review are consistent with and expand upon the evidence summarized in the previous literature. Across the studies included in our review, falls were uniformly defined as unexpected, non-violent events leading to rest on the ground or a lower level reflecting current consensus definitions used in the field. This methodological consistency facilitates comparison across studies and supports the integration of our results within the broader literature.

Similar to the findings of [9,10,13] our results indicate that fall risk arises from the interaction of multiple domains, including physical, clinical, cognitive, behavioral, and social factors. Physical determinants such as balance impairment, reduced gait speed, low physical activity, limited range of motion, malnutrition, frailty, and diminished muscle strength were consistently reported, aligning with previous evidence that mobility-related impairments represent the most robust physical and functional risk factors for falls.

The findings of [5], which emphasized the role of lower-limb strength and knee extensor torque in mobility decline, further support the role of muscular function in maintaining postural control and stability. When combined with other factors such as balance deficits, functional limitations, and frailty these muscular impairments may substantially increase the risk of falls among community-dwelling older adults.

The results of our review also converge with prior findings highlighting cognitive decline, especially executive dysfunction, as a key contributor to falls susceptibility. The identification of executive function as a relevant determinant assessed through tools such as the Trail Making Test. Executive function encompasses a set of higher-order cognitive processes, including planning, inhibition, working memory, and cognitive flexibility, that enable individuals to regulate behavior and adapt to environmental demands [29]. In older adults, impairments in executive function are particularly relevant to fall risk, as these cognitive processes are crucial for maintaining postural control, managing attention while walking, and responding to unexpected balance perturbations. Declines in executive control can reduce an individual’s ability to coordinate motor and cognitive tasks simultaneously, thereby increasing the risk of falls during complex or dual-task situations [30].

Furthermore, clinical and behavioral risk factors such as chronic diseases (e.g., cardiovascular disorders, hypertension, Parkinson’s disease), and polypharmacy were consistently associated with higher fall risk, mirroring meta-analytic findings that these may impair balance and increase orthostatic instability [9,10,13,27] and the association between frailty and chronic multimorbidity identified in our synthesis also aligns with the broader evidence base linking geriatric syndromes to mobility limitation and recurrent falls.

Complementary to these clinical domains, our results highlight the influence of social and environmental determinants, including living alone, rural residence, and limited sensory function, which may exacerbate isolation, reduce activity levels, and increase exposure to environmental hazards [9,10,27]. This evidence reinforces recent calls in the literature for a more comprehensive approach to falls risk assessment, one that integrates psychosocial, sociodemographic, and environmental contexts, alongside physical and cognitive measures.

Finally, functional performance outcomes such as SPPB < 10, gait speed < 0.8 m·s^−1^, and TUG > 12 s were consistently associated with higher fall risk, confirming the predictive validity of standardized clinical assessments widely used in gerontology research and practice [11,13]. Taken together, these findings underscore that mobility-related constructions remain one of the best clinical assessments of risk of falls in community dwelling older adults. In summary, integrating physical, cognitive, and behavioral indicators may offer a more accurate and clinically relevant framework for identifying older adults at greatest risk and guiding targeted, evidence-based prevention strategies. Collectively, the evidence supports multifactorial risk but lacks mechanistic integration. Future research should incorporate theoretically driven models that capture interacting risk pathways rather than isolated predictors.

### 4.2. Predictor Models of Falls

This review highlights the ongoing challenge of accurately predicting models of fall among community-dwelling older adults. Consistent with previous findings by [15], most prognostic models demonstrated a high risk of bias due to restrictive eligibility criteria, inconsistent falls definitions, incomplete data handling, and inadequate statistical validation. Consequently, their predictive accuracy and clinical applicability remain limited.

A major insight from this synthesis is the discrepancy between strong individual predictors (e.g., prior falls, gait speed, executive function) and weak predictive models. The overreliance on fall history reflects a conceptual limitation that the models prioritize retrodictive accuracy in recurrent falls rather than uncovering mechanisms capable of predicting first-time fallers.

The reviewed models showed wide variability in performance, with area under the curve of sensitivity values ranging from 0.49 to 0.87 [15]. Similarly, Wapp [16] identified prior falls history as the strongest single predictor of future falls, outperforming demographic, clinical, and functional measures. However, reliance on this variable alone limits the capacity to identify first-time fallers, underscoring the need for models that incorporate additional sensitive predictors.

Supporting evidence from recent studies [9,10,13] reinforces that falls risk is multifactorial, shaped by mobility impairments, comorbidities, medication use, cognitive decline, and behavioral and social factors. Future prediction models should integrate these multidimensional variables, adopt standardized outcome definitions, and apply robust validation procedures to improve accuracy, generalizability, and clinical utility in identifying older adults at greatest risk of falling.

The methodological weaknesses identified in the included studies reflect persistent and fundamental shortcomings in the field. As highlighted in previous meta-research, inconsistent and poorly justified fall definitions compromise comparability across studies, while inadequate handling of missing data introduces substantial bias into predictive estimates. Moreover, the lack of external validation and the failure to model nonlinear or interactive effects among predictors indicate a limited methodological sophistication that undermines the credibility of proposed models. The frequent reliance on homogeneous samples further restricts generalizability and raises concerns about the real-world applicability of these findings. Together, these deficiencies substantially weaken the evidentiary foundation on which current fall prediction models are built.

Moreover, most models ignore insights from computational motor control and complex systems theory, which indicate that small perturbations in gait variability, neuromuscular timing, or dual-task performance can predict instability long before overt falls occur [30,31]. Integrating these sensitive predictors may improve the detection of early decline.

### 4.3. Exercise Therapy to Prevent Falls

The exercise therapeutic approach is widely recognized as one of the most effective strategies for preventing falls among older adults [3]. The benefits of exercise therapy extend beyond improvements in balance and strength to include gains in aerobic capacity, postural control, and endurance, which collectively reduce fatigue and fall susceptibility Moreover, regular exercise contributes to the prevention of frailty, cognitive decline, and cardiovascular comorbidities. Despite these advantages, the absence of standardized guidelines regarding training volume, intensity, and progression continues to hinder the consistent implementation of exercise-based falls prevention protocols [5].

In line with previous evidence, the findings of [18] suggest that multicomponent training programs, combining strength, balance, and aerobic exercises, and single-component interventions such as balance-focused training, produce comparable improvements in physical and physiological outcomes. However, the evidence remains inconclusive regarding actual falls incidence, suggesting that functional improvement does not always translate directly into reduced falls rates. Studies such as [27] demonstrate that targeted single-component programs like Tai Chi, which emphasize dynamic balance and coordination, may be more effective in reducing falls than general multicomponent approaches. This superiority likely stems from the specificity of balance-focused stimuli, which enhances sensorimotor integration and postural control mechanisms crucial for maintaining center of mass stability.

Recent research has also highlighted the growing role of home-based and technology-supported exercise programs as accessible alternatives for older adults. Zhou and Cao [14,19] found that such interventions, particularly those incorporating digital or electronic devices, can reduce falls risk by 18–34% and support greater adherence. Nevertheless, challenges related to exercise supervision, safety, and consistency persist, underscoring the need for hybrid models that combine remote and in-person guidance. The implementation of hybrid exercise training models for falls prevention in older adults may represent a promising and pragmatic approach considering the growing aging population worldwide. Such models are particularly appropriate for highly functional community-dwelling older adults. Recent implementation research, including [32], shows that fall-prevention exercise depends not only on physiological effects but also on contextual factors influencing adoption and adherence. These insights reinforce the need for approaches that consider both efficacy and feasibility in community settings.

Specific balance exercises modalities such as Tai Chi appears to be particularly beneficial for improving functional balance, while whole-body vibration, Pilates and perturbation-based training demonstrated inconsistent or non-effects on falls rates [21,22]. Importantly, interventions that integrate cognitive and physical components may enhance outcomes for older adults with mild cognitive impairment, supporting the inclusion of dual-task or cognitively engaging exercises in future protocols [27].

Despite promising results, variability in study designs, follow-up duration, and outcome measures limits comparability across trials. Many studies rely on surrogate indicators such as balance or gait rather than recording actual fall events. Future research should adopt standardized falls definitions, longer intervention periods, and robust methodological designs to better evaluate true preventive effects.

In clinical practice, exercise prescriptions should prioritize balance, coordination, and reactive control, particularly for older adults at higher risk of falls. Integrating multifactorial, individualized, and technology-assisted programs may represent the most effective strategy for sustainable, population-level falls prevention.

### 4.4. Limitations

This review presents certain limitations that must be acknowledged. The literature search was confined to studies published in English and indexed in a limited number of databases (SCIELO, PEDro, and Web of Science), potentially excluding relevant non-English or regional research. Moreover, only peer-reviewed and open-access publications from 2020 onward were included, which may have omitted earlier evidence of methodological relevance. The considerable heterogeneity among study designs, populations, and outcome measures hindered direct comparisons and precluded quantitative synthesis. Variability in follow-up duration, and assessment methods further limited cross-study consistency. Additionally, many trials focused on surrogate indicators of falls risk rather than actual falls incidence and often featured small samples and short intervention periods, constraining generalizability. Although this review adhered to PRISMA-ScR guidelines, selection and publication biases cannot be entirely excluded.

Despite the limitations, the present study provides a comprehensive and structured synthesis of recent evidence on fall-related risk factors, predictive models of falls, and exercise therapeutic interventions among community-dwelling older adults. By integrating findings across multiple thematic domains, the review captures the multifactorial and interdependent nature of falls, reflecting the complexity of this public health issue. Additionally, the review focused exclusively on recent and peer-reviewed literature (2020–2025), allowing for the inclusion of contemporary findings that align with the latest advances in falls prediction modeling, and exercise prescription. This temporal focus enhances the relevance of the conclusions for current clinical practice.

Future research should prioritize the development of standardized, multidimensional frameworks for falls assessment and prevention in older adults, integrating physical, cognitive, behavioral, and environmental factors into predictive models. Validation of existing models through large-scale, longitudinal studies with diverse populations, including those with cognitive decline and multimorbidity, is essential. In exercise-based interventions, future trials must define standardized parameters for training volume, intensity, and progression to enable comparability and evidence-based guideline development.

### 4.5. Clinical Implications

The findings of this review underscore the need for a paradigm shift in clinical practice toward more comprehensive and individualized approaches to fall prevention. Multidimensional assessment should replace single-factor screening, with clinicians combining gait analysis, executive function, polypharmacy review, and frailty indices to capture the complex interplay of determinants underlying fall risk. Although prior fall history remains a strong predictor, it must be supplemented with sensitive indicators of early decline to improve detection of high-risk individuals. Exercise interventions should be individualized and deficit-oriented rather than based on generic fall-prevention bundles, ensuring that the prescribed training directly targets the mechanisms contributing to instability. Hybrid delivery models that integrate in-person and digital components offer promising avenues to enhance adherence and scalability. Finally, reactive balance training should be incorporated more systematically, given its ecological relevance and potential to improve real-world fall resilience.

## 5. Conclusions

According to our findings, falls among community-dwelling older adults represent a complex and multifactorial phenomenon influenced by physical, cognitive, behavioral, and environmental factors. Our results indicate that although exercise interventions are among the most effective strategies for fall prevention, the absence of standardized parameters for exercise prescription continues to hinder their comparability and clinical applicability. Furthermore, existing predictive models lack sufficient accuracy and external validation to be reliably implemented in real-world settings. Therefore, future research should prioritize the development of integrated, multidimensional frameworks that combine physical, cognitive, and behavioral predictors with personalized exercise approaches to enhance fall prevention strategies in older adults.

## Figures and Tables

**Table 1:** PRISMA-ScR Checklist.

Item	PRISMA-ScR Requirement	Reported?	Location in Manuscript
1	Identify report as scoping review	Yes	Title
2	Structured summary	Yes	Abstract
3	Rationale	Yes	Introduction
4	Objectives	Yes	Introduction
5	Protocol and registration	Yes	Methods
6	Eligibility criteria	Yes	Methods
7	Information sources	Yes	Methods
8	Search strategy	Yes	Methods + Table 1
9	Selection of sources of evidence	Yes	Methods
10	Data charting processes	Yes	Methods
11	Data items	Yes	Methods
12	Critical appraisal of sources	Not applicable	—
13	Synthesis of results	Yes	Methods
14	Selection of sources (results)	Yes	Results + Flowchart
15	Characteristics of included studies	Yes	Results + Tables
16	Critical appraisal within studies	Not applicable	—
17	Results of individual sources	Yes	Results
18	Synthesis of results	Yes	Results
19	Summary of evidence	Yes	Discussion
20	Limitations	Yes	Section 4.4
21	Conclusions	Yes	Conclusion
22	Funding	Yes	Title page

**Table 2:** SCIELO, PUBMED, and PEDro search stings.

Database	Search Strategy/Terms
PubMed	(“Accidental Falls” [Mesh] OR “falls” [tiab] OR “fall risk” [tiab] OR “fall prevention” [tiab]) AND (“Aged” [Mesh] OR “older adults” [tiab] OR “elderly” [tiab] OR “aging population” [tiab]) AND (“Risk Factors” [Mesh] OR “predictive factors” [tiab] OR “prediction model” [tiab]) AND (“Exercise Therapy” [Mesh] OR “Physical Therapy Modalities” [Mesh] OR “Rehabilitation” [Mesh] OR “physical exercise” [tiab] OR “physiotherapy” [tiab])
SCIELO	(“queda*” OR “falls” OR “fall risk” OR “fall prevention”) AND (“idoso*” OR “older adults” OR “elderly”) AND (“fatores de risco” OR “risk factors” OR “predictive factors”) AND (“exercício físico” OR “physical exercise” OR “fisioterapia” OR “physical therapy” OR “rehabilitation”)
PEDro	Population: older adults OR elderly OR aged; Intervention: physical therapy OR physiotherapy OR exercise OR rehabilitation; Outcome: falls OR fall risk OR fall prevention

**Table 3:** Summary of risk factors for falls among community-dwelling older adults.

Domain	Specific Risk Factors	Supporting Evidence
Physical and Functional	Impaired mobility; balance impairment; low physical activity and functional capacity; reduced range of motion; sarcopenia; malnutrition; bone mass loss; and frailty	[7,8,9,11]
Clinical status	Chronic comorbidities; and polypharmacy	[7,8]
Cognitive and Sensory	Advanced age (>80 years); cognitive decline; reduced or uncorrected vision; sensory impairments	[9,10,11]
Psychological and Behavioral	Fear of falling; sedentary lifestyle; smoking; alcohol consumption; and depression	[7,11]
Social	Single marital status; and rural residence	[7,8]
Functional Performance Tests	SPPB score < 10 points; gait speed < 0.8 m·s^−1^; Timed Up and Go test > 12 s	[9,12]

## Data Availability

The original contributions presented in this study are included in the article. Further inquiries can be directed to the corresponding author.
